# Assessing the risk factors and establishing multivariable prediction models for singleton macrosomia

**DOI:** 10.3389/fmed.2025.1590283

**Published:** 2025-09-24

**Authors:** Jinying Luo, Wenyan Huang, Suping Luo, Lin Deng, Lihua Lin, Qiuping Liao, Jianying Yan, Jinfu Zhou

**Affiliations:** ^1^College of Clinical Medicine for Obstetrics and Gynecology and Pediatrics, Fujian Medical University, Fujian Maternity and Child Health Hospital, Fuzhou, Fujian, China; ^2^Fujian Clinical Research Center for Maternal-Fetal Medicine, Fuzhou, Fujian, China; ^3^National Key Obstetric Clinical Specialty Construction Institution of China, Fuzhou, Fujian, China; ^4^Department of Obstetrics and Gynecology, Quanzhou Women & Children's Hospital, Quanzhou, Fujian, China; ^5^Department of Epidemiology and Health Statistics, School of Public Health, Fujian Medical University, Fuzhou, Fujian, China; ^6^Department of Healthcare, Fujian Maternity and Child Health Hospital, College of Clinical Medicine for Obstetrics & Gynecology and Pediatrics, Fujian Medical University, Fuzhou, Fujian, China; ^7^Medical Genetic Diagnosis and Therapy Center, Fujian Key Laboratory for Prenatal Diagnosis and Birth Defect, Fujian Maternity and Child Hospital College of Clinical Medicine for Obstetrics & Gynecology and Pediatrics, Fujian Medical University, Fuzhou, Fujian, China

**Keywords:** macrosomia, predictive model, pre-pregnancy body mass index, gestational weightgain, gestational diabetes mellitus, risk factors

## Abstract

**Introduction:**

Fetal macrosomia is related to adverse neonatal and maternal health outcomes. Therefore, we aimed to evaluate the risk factors for macrosomia and establish multivariable prediction models to enable early identification, prevention, and mitigation of its adverse outcomes.

**Methods:**

This retrospective case-control study included 800 singleton pregnant women who delivered in 2022 at Fujian Maternity and Child Health Hospital and Quanzhou Women and Children's Hospital. They were categorized into the macrosomia [birth weight (BW) ≥ 4,000 g, *n* = 400] and non-macrosomia (BW = 2,500–3,999 g, *n* = 400) groups according to the BW of the newborns. Prediction models in singleton fetuses during mid-to-late pregnancy and before delivery were constructed.

**Results:**

Maternal height ≥ 165 cm [odds ratio (OR) = 2.303, 95% confidence interval (CI): 1.232–4.305], pre-pregnancy overweight (OR = 2.166, 95% CI: 1.119–4.195), pre-pregnancy obesity (OR = 3.189, 95% CI: 1.020–9.968), excessive gestational weight gain in the second trimester (OR = 2.083, 95% CI: 1.250–3.470), and at least two abnormal blood glucose values in the oral glucose tolerance test (OR = 5.267, 95% CI: 1.814–15.29) were identified as risk factors for macrosomia. Additionally, maternal abdominal circumference (AC) plus fundal length ≥ 140 cm (OR = 6.283, 95% CI: 3.976–9.927), fetal biparietal diameter ≥ 10 cm (OR = 3.373, 95% CI: 1.103–10.31), fetal head circumference ≥ 35 cm (OR = 3.473, 95% CI: 1.334–9.041), and fetal AC ≥ 36 cm at pre-delivery (OR = 23.46, 95% CI: 14.81–37.16) were risk factors for macrosomia.

**Discussion:**

The construction of the macrosomia prediction model in singleton fetuses during mid-to-late pregnancy and before delivery showed a strong predictive value. This study identified key high-risk factors for macrosomia during the perinatal period. The macrosomia prediction model developed here is expected to enable early identification of macrosomia, allowing for timely interventions aimed at reducing the risk of adverse perinatal outcomes.

## 1 Introduction

Fetal macrosomia is a major concern for neonatal and maternal health that potentially leads to severe perinatal complications (1). The American College of Obstetrics and Gynecology defines fetal macrosomia as an estimated fetal weight or birth weight (BW) exceeding 4,000 g, regardless of gestational age (2). In developed countries, the incidence rate of macrosomia has recently risen from 5%−20% to 15%−25% (3). Moreover, the rapid socioeconomic development in China has significantly altered the dietary habits and lifestyles of residents, contributing to an increasing prevalence of macrosomia (4). The overall prevalence of macrosomia in China increased from 6.6% in 1996 to 8.7% in 2014 (5–7).

Numerous studies have shown a significant relationship between macrosomia and an increased risk of maternal and neonatal complications in the short and long term. Maternal complications primarily include emergency cesarean section, postpartum hemorrhage (PPH), and anal sphincter injury (8, 9). In contrast, neonatal complications involve fractures, perinatal asphyxia, cerebral hemorrhage, and brachial plexus injuries (1, 10). Macrosomia also increases the risk of metabolic diseases, including obesity, diabetes, and hypertension in adulthood (11–13). Beyond physical impacts, it may elevate psychiatric risk during adolescence, particularly among males (14). Therefore, early identification and prediction of macrosomia, followed by the implementation of preventive measures, are crucial for reducing its incidence and mitigating its adverse outcomes, which have significant scientific and practical implications.

Studies have demonstrated that macrosomia is associated with various factors, including environmental influences (15), maternal blood lipid levels (16), gestational weight gain (GWG) (17), gestational diabetes mellitus (GDM) (4), delivery history of macrosomic infants (6), pre-pregnancy body mass index (BMI) (18), parity (19), and other reproductive factors (20). Currently, fetal weight is estimated during prenatal care through maternal physical examinations or ultrasonographic measurements. However, the accuracy of macrosomia prediction using a single clinical indicator remains low. Therefore, developing a practical and accurate predictive model for macrosomia is essential. Multivariable prediction models, which are increasingly used in healthcare, estimate an individual's risk of developing a condition by incorporating multiple characteristics (21, 22). These models have been employed in obstetrics to predict preterm birth (23), small-for-gestational-age infants (24), and preeclampsia (25).

Therefore, this study aimed to evaluate the risk factors for macrosomia and establish multivariable prediction models to enable early identification, prevention, and mitigation of its adverse outcomes.

## 2 Materials and methods

### 2.1 Study design and population

This retrospective case-control study included 800 singleton pregnant women who delivered in 2022 at Fujian Maternity and Child Health Hospital and Quanzhou Women and Children's Hospital. Participants were categorized into two groups based on the BW of newborns exceeding 4,000 g—the macrosomia (case group) and non-macrosomia (control group) groups. This study was conducted in accordance with the guidelines of the Declaration of Helsinki and approved by the Ethics Review Committee of Fujian Provincial Maternity and Child Hospital, Fuzhou, China (approval number: 2023KY046). Written informed consent was obtained from the guardians of all participants after a detailed description of the study's purpose was provided.

### 2.2 Data collection

At pregnancy registration, data on maternal demographic characteristics (e.g., age, education, and occupation), anthropometric measurements (e.g., body weight, height, and blood pressure), and clinical history (e.g., parity and disease history) were recorded. Anthropometric measurements were also collected during mid and late pregnancy. Fasting blood samples and laboratory tests (e.g., routine blood checkup and blood biochemistry examination, including lipids and glucose levels) were performed at 24 weeks of gestation. Subsequently, results from the 75 g oral glucose tolerance test (OGTT) for diagnosing GDM were recorded. Data on maternal blood lipid levels, including total triglyceride (TG), total cholesterol (TC), high-density lipoprotein cholesterol (HDL-C), and low-density lipoprotein cholesterol (LDL-C), were collected during late pregnancy. The most cost-effective method for detecting fetal macrosomia is selective ultrasound scanning for all suspected cases during late pregnancy (26). Ultrasound results, including biparietal diameter (BPD), head circumference (HC), abdominal circumference (AC), and femur length (FL), were recorded. Clinical records related to delivery (e.g., delivery mode, BW, sex, and Apgar score) were retrieved.

Macrosomia was defined as a BW of ≥ 4,000 g (27). Maternal pre-pregnancy BMI was calculated based on self-reported height and weight values before conception, as provided by the participants in the questionnaire. This was categorized as low (BMI < 18.5 kg/m^2^), normal (BMI: 18.5–23.9 kg/m^2^), overweight (BMI: 24.0–27.9 kg/m^2^), and obese (BMI ≥ 28.0 kg/m^2^) according to the Chinese Nutrition Society Group standard “weight monitoring and evaluation during the pregnancy period of Chinese women” (https://www.cnsoc.org/otherNotice/392100200.html/T/CNSS-009-2021). GDM was diagnosed following the International Association of Diabetes and Pregnancy Study Group criteria, using 75 g 2-h OGTT: fasting glucose ≥ 5.1 mmol/L, 1-h glucose ≥ 10.0 mmol/L, or 2-h glucose ≥ 8.5 mmol/L (28).

GWG was calculated as the difference between the weight at delivery and the pre-pregnancy self-reported weight. Excessive GWG was considered when the total weight gain recommendations of the Chinese Nutrition Society Group standard were exceeded. These recommendations are as follows: (1) underweight: 11.0–16.0 kg, (2) normal weight: 8.0–14.0 kg, (3) overweight: 7.0–11.0 kg, and (4) obese: 5.0–9.0 kg. The recommended GWG in early pregnancy is 2 kg. Furthermore, the normal rate of GWG during mid and late pregnancy for underweight, normal weight, overweight, and obese women is 0.37–0.56, 0.26–0.48, 0.22–0.37, and 0.15–0.30 kg/week, respectively (https://www.cnsoc.org/otherNotice/392100200.html/T/CNSS-009-2021). High maternal TG, TC, and LDL-C, as well as fetal BPD, HC, AC, and FL, were defined as values ≥ 95th percentile. Low maternal HDL-C was defined as values ≤ 5th percentile. High maternal height were defined as ≥ 165 cm (29, 30).

### 2.3 Statistical analyses

All statistical analyses were performed using IBM SPSS Statistics for Windows, version 26.0 (IBM Corp., Armonk, N.Y., USA). Normally distributed continuous variables were expressed as mean ± standard deviation, with independent samples used to compare both groups and one-way analysis of variance for multiple groups. Non-normally distributed data were presented as median and interquartile range, with non-parametric tests (Mann–Whitney *U* test) employed for data with unequal variances. Categorical data were presented as frequency (*n*) and percentage (%), with chi-square or Fisher's exact test used for intergroup comparisons. Univariate logistic regression was performed to assess the relationship between macrosomia and individual predictor variables. Multivariate logistic regression analysis was performed to build multivariate prediction models in singleton fetuses during mid-to-late pregnancy and before delivery using all relevant predictors. Final predictors for the model were selected using bidirectional elimination with stepwise backward regression, starting with a *P-*value threshold of < 0.15. The final predictor selection for the models involved evaluating all combinations of candidate predictors with entry and retention criteria set at *P*-values of 0.40 and 0.20, respectively. Multivariable prediction models were evaluated using the Hosmer–Lemeshow goodness-of-fit tests. Subsequently, the models' predictive performance was assessed using receiver operating characteristic (ROC) curves. The predictive accuracy was determined at the model probability cut-off corresponding to the maximum Youden's index. Two-tailed tests were used, with *P* < 0.05 considered statistically significant.

## 3 Results

### 3.1 Maternal and neonatal characteristics of the 800 participants included in the case-control study

Overall, 800 women with singleton pregnancies were included in this study. Table 1 shows the maternal and neonatal characteristics. No significant differences in the mean maternal age, proportion of pregnant women aged >35 years, proportion of assisted reproductive technology conception, or mean gestational age at delivery were observed between the macrosomia and non-macrosomia groups (*P* > 0.05). The mean maternal height, pre-pregnancy BMI, GWG, and maternal fundal length at pre-delivery were higher in the macrosomia group than in the non-macrosomia group (*P* < 0.05). Additionally, the proportions of pre-pregnancy overweight and obese women, with gravidity of ≥2 times and parity of ≥1 times in the macrosomia group, were higher than those in the non-macrosomia group. Although no significant difference in the mean GWG rates during the first, second, and third trimesters was observed between the macrosomia and non-macrosomia groups, the proportions of patients with excessive GWG rates in each trimester were higher in the macrosomia group than in the non-macrosomia group. The mean BW of newborns and proportions of male infants were significantly higher in the macrosomia group than in the non-macrosomia group (*P* < 0.001). Conversely, both groups showed no significant differences in newborn birth height.

**Table 1:** Maternal and neonatal characteristics of the 800 cases from Fujian Maternity and Child Health Hospital and Quanzhou Women and Children's Hospital.

**Variables**	**Macrosomia group (*N* = 400) Mean (SD)/median or *n* (%)**	**Non-macrosomia group (*N* = 400) Mean (SD)/median or *n* (%)**	***t*/*Z*/χ^2^**	***P*-value**
**Maternal characteristics**				
**Education level**			2.758	0.097
Junior college or less	141 (25.3)	119 (29.8)		
University degree or above	259 (74.7)	281 (70.3)		
**Maternal age (year)**				1.00
< 35	341 (85.3)	341 (85.3)		
≥35	59 (14.7)	59 (14.7)		
**Categories of gestational age at delivery (weeks)**			52.86	< 0.001
< 40	213 (53.3)	112 (28.0)		
≥40	187 (46.8)	288 (72.0)		
**Categories of maternal height (cm)**			9.853	0.002
< 165	292 (73.0)	329 (82.3)		
≥165	108 (27.0)	71 (17.8)		
Assisted reproductive technology conception	28 (7.0)	34 (8.5)	0.629	0.428
**Gravidity**			8.851	0.003
1	118 (29.5)	158 (39.5)		
≥2	282 (70.5)	242 (60.5)		
**Parity**			4.514	0.034
0	174 (43.5)	204 (51.0)		
≥1	226 (56.5)	196 (49.0)		
Pre-pregnancy BMI (kg/m^2^)	22.27 (20.32, 24.65)	20.70 (19.08, 22.63)	7.654	< 0.001
**Categories of pre-pregnancy BMI**			44.53	< 0.001
Underweight (< 18.5)	29 (7.2)	63 (15.8)		
Normal weight (18.5–24.9)	242 (60.5)	281 (70.3)		
Overweight (25.0–29.9)	99 (24.8)	45 (11.3)		
Obesity (≥30)	30 (7.5)	11 (2.8)		
Weight at pre-pregnancy (kg)	58.75 (53.0, 65.0)	53.25 (48.63, 58.0)	8.615	< 0.001
Weight at delivery (kg)	75.0 (67.5, 81.73)	67.0 (62.0, 72.0)	11.04	< 0.001
Gestational age of the last ultrasound scanning (week)	39 (38, 40)	38 (38, 39)	7.135	< 0.001
Maternal abdominal circumference at pre-delivery (cm)	104 (100, 108)	98 (95, 102)	13.19	< 0.001
Maternal fundal length at pre-delivery (cm)	36.5 (35, 38)	34 (33, 35)	18.01	< 0.001
Gestational weight gain (GWG; kg)	15.0 (12.5, 19.0)	14.0 (11.0, 16.5)	5.265	< 0.001
GWG rate during the first trimester (kg/week) GWG	0.12 (0.0, 0.25)	0.02 (−0.05, 0.15)	4.935	< 0.001
**Categories of GWG rate during the first trimester**			20.53	< 0.001
Normal	43 (18.4)	45 (19.5)		
Over	109 (46.6)	63 (27.3)		
Under	82 (35.0)	123 (53.2)		
GWG rate during the second trimester(kg/week) GWG	0.54 (0.43, 0.65)	0.49 (0.38, 0.60)	2.354	0.019
**Categories of GWG rate during the second trimester**			12.245	0.002
Normal	46 (27.7)	67 (42.4)		
Over	115 (69.3)	80 (50.6)		
Under	5 (3.0)	11 (7.0)		
GWG rate during the third trimester (kg/week) GWG	0.49 (0.36, 0.63)	0.5 (0.38, 0.64)	0.473	0.636
**Categories of GWG rate during the third trimester**			0.408	0.815
Normal	70 (33.0)	72 (34.8)		
Over	122 (57.5)	113 (54.6)		
Under	20 (9.4)	22 (10.6)		
Gestational age at delivery (week)	40 (39, 40)	39 (38, 40)	6.351	< 0.001
GDM	85 (21.3)	85 (21.3)	7.241	0.007
**Abnormal blood glucose value of OGTT**			9.783	0.002
≤ 1	356 (89.0)	380 (95.0)		
≥2	44 (11.0)	20 (5.0)		
Polyhydramnios	34 (8.5)	7 (1.8)	18.74	< 0.001
**Neonatal characteristics**			24.22	< 0.001
Birth weight (kg)	4.1 (4.03, 4.25)	3.3 (3.05, 3.5)		
Birth length (cm)	52 (51, 52)	50 (49, 50)	19.05	< 0.001
**Gender**			14.54	< 0.001
Male	279 (69.8)	227 (56.8)		
Female	121 (30.3)	173 (43.3)		

BMI, body mass index; GWG, gestational weight gain; GDM, gestational diabetes mellitus; OGTT, oral glucose tolerance test.

### 3.2 Comparisons of blood glucose and lipid levels between the macrosomia and non-macrosomia groups in late pregnancy

To assess the effect of maternal glucose and lipid levels on fetal weight during pregnancy, blood glucose levels in the OGTT and blood lipids in late pregnancy were compared between the macrosomia and non-macrosomia groups.

Pregnant women in the macrosomia group exhibited a higher GDM incidence, a greater proportion of at least two abnormal OGTT values, and higher mean blood glucose levels at OGTT0 and OGTT2 than those in the non-macrosomia group (*P* < 0.05). In late pregnancy, the mean HDL-C levels and proportions of pregnant women with HDL-C ≤ 1.27 mmol/L were significantly lower in the macrosomia group than in the non-macrosomia group. However, no significant differences were observed in TG, TC, and LDL-C levels between the two groups (*P* > 0.05; Table 2).

**Table 2:** Comparisons of the level of blood glucose of OGTT and blood lipids at late pregnancy between the macrosomia and non-macrosomia groups.

**Variables**	**Macrosomia group (*N* = 400) Mean (SD)/median or *n* (%)**	**Non-macrosomia group (*N* = 400) Mean (SD)/median or *n* (%)**	***t*/*Z*/χ^2^**	***P*-value**
GDM	85 (21.3)	85 (21.3)	7.241	0.007
**Abnormal blood glucose value of OGTT**				
≤ 1	356 (89.0)	380 (95.0)	9.783	0.002
≥2	44 (11.0)	20 (5.0)		
OGTT0 (mmol/L)	4.64 ± 0.47	4.42 ± 0.36	7.603	0.001
OGTT1 (mmol/L)	8.34 ± 1.77	7.96 ± 1.56	3.216	0.066
OGTT2 (mmol/L)	7.12 ± 1.50	6.79 ± 1.29	3.305	0.029
TG (mmol/L)	4.34 ± 2.04	3.78 ± 1.72	4.221	0.090
**Categories of TG (mmol/L)**				
< 7.67	377 (94.3)	382 (95.5)	0.643	0.423
≥7.67	23 (5.8)	18 (4.5)		
TC (mmol/L)	6.45 ± 1.22	6.41 ± 1.12	0.588	0.181
**Categories of TC (mmol/L)**				
< 8.57	377 (94.3)	381 (95.3)	0.402	0.526
≥8.57	23 (5.8)	19 (4.8)		
HDL (mmol/L)	1.78 ± 0.53	1.89 ± 0.40	3.121	0.002
**Categories of HDL (mmol/L)**				
< 1.27	32 (8.0)	11 (2.8)	10.838	0.001
≥1.27	368 (92.0)	389 (97.3)		
LDL (mmol/L)	3.29 ± 1.08	3.34 ± 0.86	0.700	0.484
**Categories of LDL (mmol/L)**				
< 5.02	375 (93.8)	383 (95.8)	1.608	0.205
≥5.02	25 (6.3)	17 (4.3)		

GDM, gestational diabetes mellitus; OGTT, oral glucose tolerance test; TG, total triglycerides; TC, total cholesterol; HDL, high-density lipoprotein; LDL, low-density lipoprotein.

### 3.3 Comparisons of fetal growth indicators at the last ultrasound examination before delivery between the macrosomia and non-macrosomia groups

To evaluate the predictive value of BPD, HC, AC, and FL at the last ultrasound examination before delivery for macrosomia, these measurements were compared between the two groups. The macrosomia group had higher mean values for BPD, HC, AC, and FL than the non-macrosomia group. Additionally, the macrosomia group exhibited significantly higher proportions of fetuses with BPD ≥ 10 cm, HC ≥ 35 cm, AC ≥ 36 cm, and FL ≥ 7.5 cm than the non-macrosomia group (Table 3).

**Table 3:** Comparisons of fetal growth indicators at the last ultrasound examination before delivery between the macrosomia and non-macrosomia groups.

**Variables**	**Macrosomia group (*N* = 400) Mean (SD)/median or *n* (%)**	**Non-macrosomia group (*N* = 400) Mean (SD)/median or *n* (%)**	***t*/*Z*/χ^2^**	***P*-value**
BPD (cm)	9.60 (9.36,9.80)	9.30 (9.07,9.50)	11.546	< 0.001
**Categories of BPD (cm)**			41.514	< 0.001
< 10	346 (86.5)	394 (98.5)		
≥10	54 (13.5)	6 (1.5)		
HC (cm)	34.00 ± 1.02	32.86 ± 1.15	14.81	< 0.001
**Categories of HC (cm)**			44.506	< 0.001
< 35	335 (83.8)	390 (97.5)		
≥35	65 (16.3)	10 (2.5)		
AC (cm)	36.79 ± 1.46	33.85 ± 1.67	26.57	< 0.001
**Categories of AC (cm)**			385.962	< 0.001
< 36	91 (22.8)	366 (91.5)		
≥36	309 (77.3)	34 (8.5)		
FL (cm)	7.31 ± 0.26	7.07 ± 0.29	12.24	< 0.001
**Categories of FL (cm)**			50.486	< 0.001
< 7.5	290 (72.5)	367 (91.8)		
≥7.5	110 (27.5)	33 (8.3)		

BPD, biparietal diameter; HC, head circumference; AC, abdominal circumference; FL, femur length.

### 3.4 Comparison of pregnancy outcomes between the macrosomia and non-macrosomia groups

Intraoperative or intrapartum hemorrhage and PPH are severe complications associated with the delivery of macrosomic infants. PPH incidence rates, intraoperative or intrapartum hemorrhage volume, and PPH volume within 120 min post-delivery were higher in the macrosomia group than in the non-macrosomia group. Women who delivered macrosomic infants had an increased risk of prenatal anemia, cesarean section, shoulder dystocia, and need for the administration of potent oxytocin. However, no significant differences were observed in the incidence rates of neonatal jaundice, neonatal asphyxia, or hospitalization in the neonatal intensive care unit between the two groups. These results are presented in Table 4.

**Table 4:** Comparisons of the pregnancy outcomes between the macrosomia and non-macrosomia groups.

**Variables**	**Macrosomia group (*N* = 400) Mean (SD)/median or *n* (%)**	**Non-macrosomia group (*N* = 400) Mean (SD)/median or *n* (%)**	***t*/Z/χ^2^**	***P*-value**
**Mother**				
**Delivery mode**			48.86	< 0.001
Cesarean section	210 (52.5)	113 (28.2)		
Vaginal delivery	190 (47.5)	287 (71.8)		
Intraoperative or intrapartum hemorrhage volume (ml)	255 (145, 400)	122.5 (100, 229.5)	10.037	< 0.001
**Postpartum hemorrhage**			5.417	0.020
Yes	17 (4.3)	6 (1.5)		
No	383 (95.8)	394 (98.5)		
PPH volume at 60 min after delivery (ml)	10 (10, 20)	10 (10, 20)	0.335	0.737
PPH volume at 90 min after delivery (ml)	10 (5, 10)	10 (5, 10)	0.705	0.481
PPH volume at 120 min after delivery (ml)	9 (5, 10)	8 (5, 10)	0.188	0.851
PPH volume during 120 min after delivery (ml)	300 (170, 425)	150 (120, 300)	9.736	< 0.001
**Prenatal anemia**			4.392	0.036
Yes	56 (14.0)	37 (9.3)		
No	344 (86.0)	363 (90.8)		
**Shoulder dystocia**			11.15	0.001
Yes	11 (2.8)	0 (0.0)		
No	389 (97.3)	400 (100.0)		
**The use of strong oxytocin**			41.53	< 0.001
Yes	192 (48.0)	104 (26.0)		
No	208 (52.0)	296 (74.0)		
**Newborn**				
**Neonatal jaundice**				
Yes	75 (18.8)	68 (17.0)		
No	325 (81.3)	332 (83.0)		
**Hospitalization treatment in NICU**			0.434	0.510
Yes	50 (12.5)	44 (11.0)		
No	350 (87.5)	356 (89.0)		
**Neonatal asphyxia**			0.704	0.402
Yes	8 (2.0)	5 (1.3)		
No	392 (98.0)	395 (98.8)		

PPH, postpartum hemorrhag; NICU, neonatal intensive care unit.

### 3.5 Logistic regression analysis of the risk factors for macrosomia and construction of the macrosomia prediction model

#### 3.5.1 Construction of the macrosomia prediction model in singleton fetuses during mid-to-late pregnancy

Multiple logistic regression analysis was conducted to identify risk factors for macrosomia and develop a predictive model in singleton fetuses during mid-to-late pregnancy, with macrosomia as the dependent variable. Statistically significant variables from univariate regression analyses in early and mid-pregnancy were included for the multivariate logistic regression analysis, such as maternal height, pre-pregnancy BMI, GWG rates in the first and second trimesters, GDM, and abnormal blood glucose levels, as shown in Table 5. The results showed that macrosomia was significantly associated with maternal height ≥ 165 cm [odds ratio (OR): 2.303, 95% confidence interval (CI): 1.232–4.305], pre-pregnancy overweight (OR: 2.166, 95% CI: 1.119–3.83), pre-pregnancy obesity (OR: 3.189, 95% CI: 1.020–9.968), excessive GWG in the second trimester (OR: 2.083, 95% CI: 1.250–3.470), and at least two abnormal blood glucose values in the OGTT (OR: 5.267, 95% CI: 1.814–15.29; Supplementary Table S1). Based on the multiple regression analysis, the prediction model was defined as follows: logit(*P*) = −0.731 + 0.834 × maternal height + 0.039 × pre-pregnancy underweight + 0.773 × pre-pregnancy overweight + 1.160 × pre-pregnancy obesity + 0.374 × excessive GWG in the first trimester −0.561 × insufficiently excessive GWG in the first trimester + 0.734 × excessive GWG in the second trimester −0.568 × insufficiently excessive GWG in the second trimester + 1.661 × at least two abnormal OGTT values.

**Table 5:** Multivariate logistic regression analysis results of risk factors in early and mid-pregnancy of macrosomia.

**Variables**	**β**	***SE***	***Wald***	***P*-value**	***OR (95% CI)***
**Height of mother (cm)**					
< 165	Ref.				
≥165	0.834	0.319	6.833	0.009	2.303 (1.232, 4.305)
**Pre-pregnancy BMI (kg/m** ^2^ **)**					
Normal (18.5–24.9)	Ref.				
Underweight (< 18.5)	0.039	0.397	0.010	0.921	1.040 (0.477, 2.266)
Overweight (25.0–29.9)	0.773	0.337	5.254	0.022	2.166 (1.119, 4.195)
Obesity (≥30)	1.160	0.581	3.977	0.046	3.189 (1.020, 9.968)
**GWG rate during the first trimester (kg/week)**					
Normal	Ref.				
Over	0.374	0.337	1.233	0.267	1.454 (0.751, 2.814)
Under	−0.561	0.323	3.026	0.082	0.571 (0.303, 1.074)
**GWG rate during the second trimester (kg/week)**					
Normal	Ref.				
Over	0.734	0.261	7.928	0.005	2.083 (1.250, 3.470)
Under	−0.568	0.645	0.778	0.378	0.566 (0.160, 2.004)
**Abnormal blood glucose value of OGTT**					
≤ 1	Ref.				
≥2	1.661	0.544	9.335	0.002	5.267 (1.814, 15.29)
Constant	−0.731	0.313	5.448	0.020	

BMI, body mass index; GWG, gestational weight gain; OGTT, oral glucose tolerance test.

**Figure 1:**
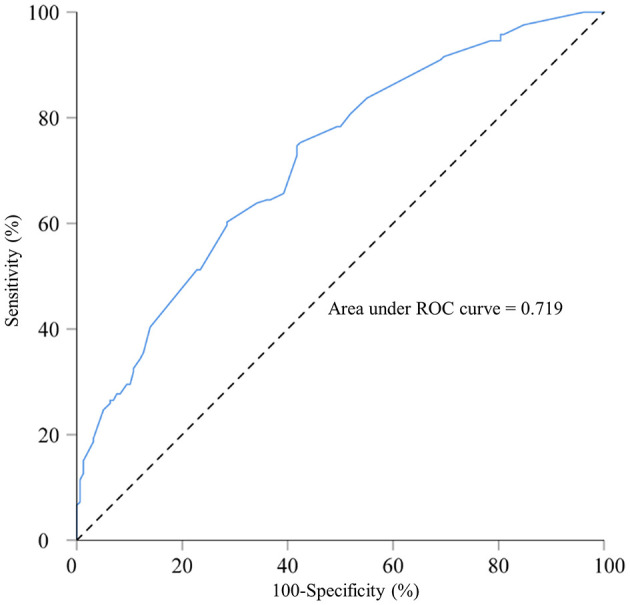
Receiver operating characteristic curve for the macrosomia prediction model in singleton fetuses during mid-to-late pregnancy; area under the curve: 0.719. A total of 800 singleton pregnant women who delivered in 2022 at Fujian Maternity and Child Health Hospital and Quanzhou Women and Children's Hospital.

ROC curve analysis was performed to examine the model's predictive performance. The area under the ROC curve (AUC) for predicting macrosomia was 0.719 (Figure 1). Additionally, the sensitivity, specificity, Youden index, positive predictive value (PPV), and negative predictive value (NPV) were 75.3, 57.6%, 0.329, 65.1, and 68.9%, respectively (Table 6). The Hosmer–Lemeshow test indicated no significant difference in the calibration of the model (Hosmer–Lemeshow χ^2^ = 3.534, *P* = 0.897), suggesting strong agreement between the model predictions and observed data.

**Table 6:** Receiver operating characteristic curve for the macrosomia prediction model in singleton fetuses during mid-to-late pregnancy and before delivery.

**Prediction model**	**AUC**	**AUC (95% CI)**	**Sensitivity**	**Specificity**	**Youden's index**	**PPV**	**NPV**	**Cutoff value**
Prediction model during mid-to-late pregnancy	0.719	0.664–0.773	75.3%	57.6%	0.329	65.1%	68.9%	0.4164738
Prediction model before delivery	0.915	0.894–0.935	82%	90.7%	0.727	89.9%	83.4%	0.5617971

AUC, area under the curve; PPV, positive predictive value; NPV:negative predictive value.

#### 3.5.2 Construction of macrosomia prediction model in singleton fetuses before delivery

To guide the timing, mode of delivery, and related risks of macrosomia, we performed a multiple logistic regression analysis to validate the risk factors for macrosomia. Additionally, we developed a prediction model for macrosomia in singleton fetuses before delivery. We incorporated the significant factors identified from univariate regression analyses during late pregnancy for further multivariate logistic regression analysis, as shown in Table 7. A significant association was found between macrosomia and maternal height ≥ 165 cm (OR: 1.729, 95% CI: 1.232–4.305), gestational age at delivery ≥ 40 weeks (OR: 1.996, 95% CI: 1.284–3.104), at least two abnormal OGTT values (OR: 5.267, 95% CI: 1.814–15.29), maternal AC plus fundal length at pre-delivery ≥ 140 cm (OR: 6.283, 95% CI: 3.976–9.927), fetal BPD ≥ 10 cm (OR: 3.373, 95% CI: 1.103–10.31), fetal HC ≥ 35 cm (OR: 3.473, 95% CI: 1.334–9.041), and fetal AC ≥ 36 cm (OR: 23.46, 95% CI: 14.81–37.16; Supplementary Table S2). The prediction model, derived from the multiple regression analysis, is as follows: logit(*P*) = −2.465 + 0.547 × maternal height + 0.691 × gestational age at delivery ≥ 40 weeks + 1.065 × at least two abnormal OGTT values + 1.838 × maternal AC plus fundal length at pre-delivery ≥ 140 cm + 1.216 × fetal BPD ≥ 10 cm +1.245 × fetal HC ≥ 35 cm + 3.155 × AC ≥ 36 cm.

**Table 7:** Multivariate logistic regression analysis results of risk factors at late pregnancy before delivery of macrosomia.

**Variables**	**β**	***SE***	***Wald***	***P*-value**	***OR* (95% *CI*)**
**Maternal height (cm)**					
< 165	Ref.				
≥165	0.547	0.258	4.072	0.034	1.729 (1.042, 2.869)
**Gestational age at delivery (weeks)**					
< 40	Ref.				
≥40	0.691	0.225	5.805	0.002	1.996 (1.284, 3.104)
**Abnormal blood glucose value of OGTT**					
≤ 1	Ref.				
≥2	1.661	0.544	9.335	0.002	5.267 (1.814, 15.29)
**Maternal abdominal circumference plus fundal length at**					
**pre-delivery (cm)**					
< 139	Ref.				
≥140	1.838	0.233	32.043	< 0.001	6.283 (3.976, 9.927)
**Fetal BPD (cm)**					
< 10	Ref.				
≥10	1.216	0.570	4.404	0.033	3.373 (1.103, 10.31)
**Fetal HC (cm)**					
< 35	Ref.				
≥35	1.245	0.488	4.631	0.011	3.473 (1.334, 9.041)
**Fetal AC (cm)**					
< 36	Ref.				
≥36	3.155	0.235	68.219	< 0.001	23.46 (14.81, 37.16)
Constant	−2.465	0.198	67.708	< 0.001	

OGTT, oral glucose tolerance test; BPD, biparietal diameter; HC, head circumference; AC: abdominal circumference.

**Figure 2:**
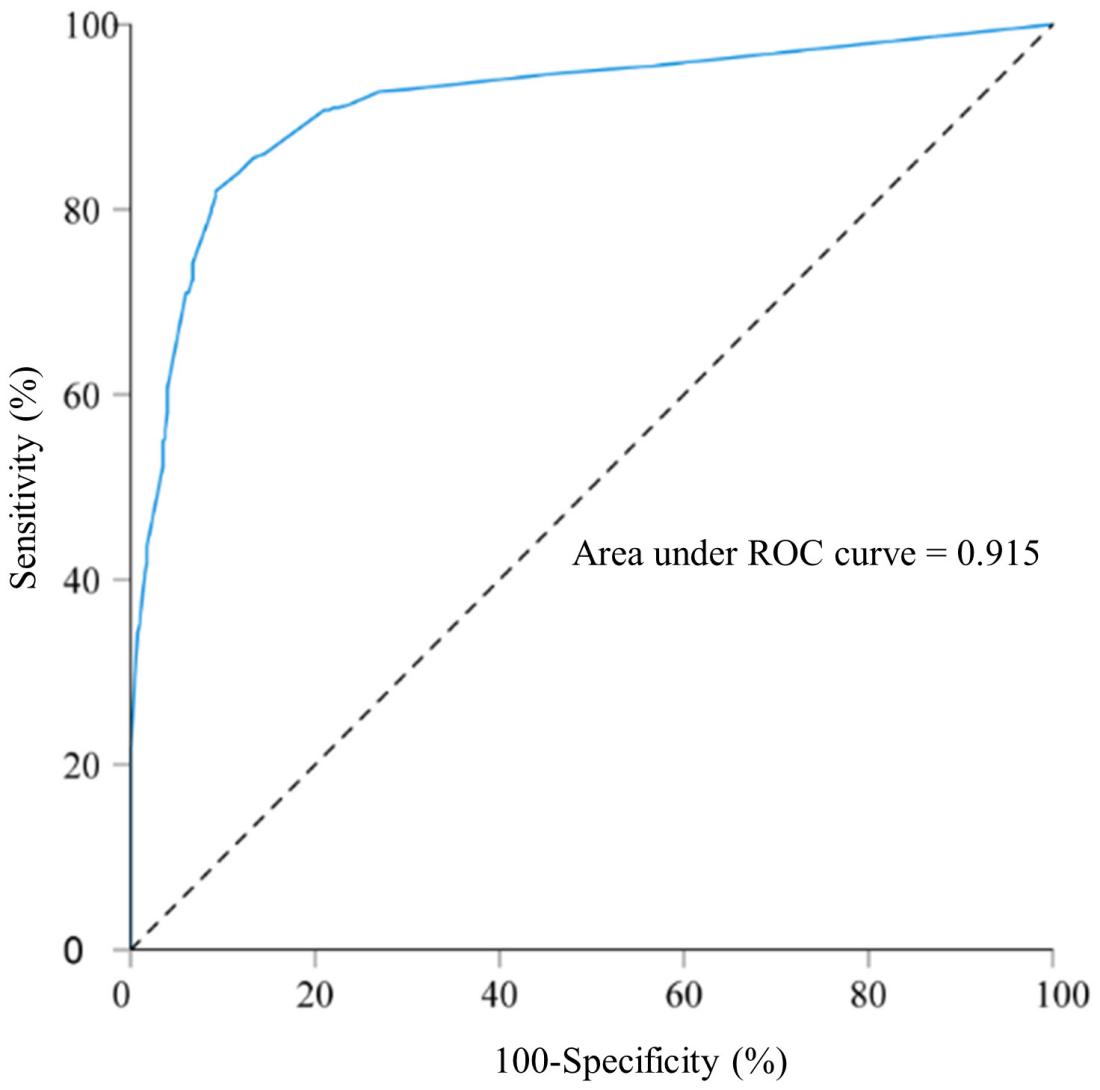
Receiver operating characteristic curve for the macrosomia prediction model in singleton fetuses before delivery; area under the curve: 0.915. A total of 800 singleton pregnant women who delivered in 2022 at Fujian Maternity and Child Health Hospital and Quanzhou Women and Children's Hospital.

ROC curve analysis was conducted to assess the model's predictive capability. The AUC for predicting macrosomia was 0.915 (Figure 2). Additionally, the sensitivity, specificity, Youden index, PPV, and NPV were 82.0%, 90.7%, 0.727, 89.9%, and 83.4%, respectively (Table 6). The Hosmer–Lemeshow test showed no significant difference in the calibration of the model (Hosmer–Lemeshow χ^2^ = 10.99, *P* = 0.089), indicating a strong agreement between the model and observed data.

## 4 Discussion

Macrosomia arises from the complex interaction of various environmental and genetic factors, thereby making the identification of risk factors and prediction more challenging. In clinical practice, these factors are categorized as unchangeable or changeable. Unchangeable factors include gravidity, parity, maternal height, and fetal sex, whereas changeable factors encompass pre-pregnancy BMI, gestational age before delivery, and GDM (31, 32). In this population-based case-control study, the relationship between macrosomia and 20 maternal and seven fetal characteristics was investigated to establish predictive models for fetal macrosomia. Maternal height ≥ 165 cm, pre-pregnancy overweight/obesity, excessive GWG during the second trimester, at least two abnormal blood glucose values on OGTT ≥ 2, gestational age at delivery ≥ 40 weeks, and maternal AC plus fundal length at pre-delivery ≥ 140 cm were identified as independent risk factors for macrosomia. The predictive model for macrosomia in singleton fetuses during mid-to-late pregnancy and before delivery showed high predictive performance.

Previous studies have revealed comparable associations between macrosomia and pre-pregnancy overweight/obesity. However, inconsistencies exist in the ORs compared with those of a normal pre-pregnancy BMI. Pre-pregnancy obesity has been associated with a 7.69-fold higher risk of macrosomia (18). Studies have demonstrated that women with obesity face a 1.5–2.3 times greater risk of macrosomia than those with a normal BMI (33, 34). In this study, we observed that women with pre-pregnancy obesity and overweight have a 3.189-fold and 2.166-fold higher risk of macrosomia, respectively, than those with a normal BMI. Excessive GWG also increases the risk of macrosomia, as confirmed by previous research (17, 35). The GWG rate during the first and second trimesters was higher in the macrosomia group than in the non-macrosomia group. Several studies have revealed GDM as an independent risk factor for macrosomia (4, 18, 36). Elevated blood sugar levels in pregnant women with GDM can be transferred to the fetus through the placenta. Since fetal pancreatic function is inadequate and maternal insulin cannot cross the placental barrier, the fetus experiences sustained elevated blood sugar levels. The synergistic effects of hyperinsulinemia and hyperglycemia in the fetus lead to enhanced glucose utilization and increased synthesis of fat and protein tissues. Reportedly, managing blood glucose levels in pregnant women with GDM through medications or dietary interventions can decrease the likelihood of macrosomia by 73% (37). Macrosomia rates in pregnant women with GDM and well-controlled blood sugar levels are comparable to those in pregnant women with normal blood sugar levels (38). Our study confirms the association between macrosomia and other risk factors, including maternal height, fetal sex (male), and gestational age ≥ 40 weeks. These findings align with those of previous studies (39–41).

Maternal blood lipid levels—TC, TG, HDL-C, and LDL-C— increase significantly at the beginning of the 12th week of gestation, particularly during the second and third trimesters (42, 43). Previous cohort studies have revealed that elevated maternal TG and reduced HDL-C levels in late pregnancy are associated with an increased risk of macrosomia (16, 44). In this study, mean HDL-C levels and the proportions of pregnant women with HDL-C ≤ 1.27 mmol/L were significantly lower in the macrosomia group than in the non-macrosomia group. However, no significant differences were observed in TG, TC, or LDL-C levels between the two groups. The discrepancies between our findings and those of earlier studies may be due to variations in methodology. All participants in this study were from the same region, ensuring comparability between the case and control groups regarding prenatal care quality and eliminating the influence of ethnic/racial factors.

While the effect of maternal obesity and overweight on excessive BW is well-known, their significant role in predicting the risk of macrosomia underscores the importance of improving the nutritional status of women to facilitate timely interventions during early and mid-pregnancy, thereby mitigating the risk of adverse neonatal outcomes. Maternal overweight or obesity is also a well-documented risk factor for GDM, which further increases the likelihood of fetal macrosomia. To clarify the relevant high-risk factors and their predictive value for macrosomia, a prediction model based on maternal height, pre-pregnancy underweight, pre-pregnancy overweight, pre-pregnancy obesity, excessive GWG during the first and second trimesters, and at least two abnormal OGTT values was developed in this study. The model achieved an AUC value of 0.719, with sensitivity, specificity, and Youden index of 75.3, 57.6%, and 0.329, respectively, indicating its predictive value. Monitoring and managing maternal weight and blood sugar levels during pre-pregnancy, early pregnancy, and mid-pregnancy is crucial for reducing the risk of macrosomia. The sensitivity and specificity are relatively low, which is mainly related to the types of variables included in the model. Specifically, this model is established based on the clinical and metabolic characteristics of the mother during the second trimester and does not incorporate the ultrasound indicators from the third trimester of pregnancy; therefore, its predictive ability is limited. Despite this, the model can identify some high-risk groups in the second trimester of pregnancy and has a certain clinical early warning value. We plan to incorporate more biological and imaging indicators in future studies and validate them in multi-center cohorts to further enhance the model's predictive performance.

Developing a predictive model for macrosomia before delivery is crucial for guiding the timing and mode of delivery. Therefore, creating efficient tools to alert healthcare professionals to the potential risks of accelerated fetal growth and macrosomia is crucial. Macrosomia was previously predicted using ultrasound and maternal physical examination (45). However, ultrasound and clinical measurements have limitations in accurately predicting newborn weight individually (2, 46, 47). In this study, we combined BPD, HC, AC, and FL from the last ultrasound examination with clinical data from pregnant women to predict macrosomia before delivery. The model achieved an AUC value of 0.915, with sensitivity, specificity, and Youden index of 82.0, 90.7%, and 0.727, respectively, indicating its high predictive value. Consequently, integrating this predictive model into clinical practice can reduce the incidence of delivery complications.

Regarding clinical application, in mid-pregnancy—especially after completing the OGTT—we can use a prediction model to predict the risk of macrosomia, and subsequently make some interventions (e.g., dietary counseling, closer glucose monitoring, ultrasound follow-ups) to prevent macrosomia at the time of delivery. When approaching the delivery time, the model can be used again before delivery to further evaluate the risk of macrosomia and decide the mode of delivery. For instance, a patient was 166 cm tall, weighed 68 kg before pregnancy, and had a pre-pregnancy BMI of 24.68 kg/m^2^. The weight gain rate was normal in both the first and second trimesters. Additionally, the OGTT at 24 weeks was 5.2–10.5–8.1 mmol/L. The patient underwent diet intervention and engaged in exercise to control the blood glucose to normal, and the GWG was 12 kg. Maternal AC plus fundal length at pre-delivery was 138 cm. The ultrasound result at 40 weeks of gestation indicated BPD, HC, AC, and FL of 9.8, 35.5, 35.5, and 7.4 cm, respectively. Notably, the patient vaginally delivered a baby weighing 3.5 kg without complications. The rate of macrosomia at 24 weeks of gestation was 92.7%, according to the equation: logit(*P*) = −0.731 + 0.834 × 1 + 0.039 × 0 + 0.773 × 1 + 1.160 × 0 + 0.374 × 0 – 0.561 × 0 + 0.734 × 0 – 0.568 × 0 + 1.661 × 1 = 2.54. Furthermore, the rate of macrosomia at 39 weeks of gestation is 74.7%, based on the equation: logit(*P*) = −2.465 + 0.547 × 1 + 0.691 × 1 + 1.065 × 1 + 1.838 × 0 + 1.216 × 0 + 1.245 × 1 + 3.155 × 0 = 1.08. This implies that when the rate of macrosomia is significantly high in mid-pregnancy, interventions should be implemented to decrease the rate before delivery, thereby increasing the likelihood of a successful vaginal birth.

This study has some limitations. First, as a retrospective case-control study, it is inherently subject to selection bias. Second, the study focused exclusively on the Chinese population due to ethnic differences, which may limit the generalizability of its findings to other racial groups. Third, the predictive model was derived and tested using a two-center dataset of Fujian province, without external validation. Additionally, the study did not include data on the history of macrosomia or the dietary intake of pregnant women, which could increase the risk of macrosomia. Therefore, future research should focus on several key areas to deepen the understanding of macrosomia. These include conducting external validation in independent cohorts of multiple centers, different regions, and different populations to comprehensively evaluate the universality and clinical application value of the model, as well as exploring additional risk factors unaddressed in this study, such as maternal diet, lifestyle factors, and environmental exposures.

In conclusion, this study identifies key high-risk factors for macrosomia during the perinatal period. We also developed a macrosomia prediction model in singleton fetuses during mid-to-late pregnancy and before delivery. ROC curve analysis showed that the model had a strong predictive value for macrosomia. We expect that this model will help doctors predict the occurrence of macrosomia early and implement interventions to mitigate adverse perinatal outcomes.

## Data Availability

The original contributions presented in the study are included in the article/Supplementary material, further inquiries can be directed to the corresponding authors.
