# Supplementary material for: Assessing the risk factors and establishing multivariable prediction models for singleton macrosomia
Source: Front Med (Lausanne). 2025 Sep 24;12:1590283. doi: 10.3389/fmed.2025.1590283 (PMC12504509; doi:10.3389/fmed.2025.1590283)
Supplement: Supplementary file 1 [file Table_1.docx]

Supplementary Material

# Supplementary Tables

**Table S1.** Logistic regression analysis of risk factors for macrosomia during mid-to-late pregnancy

| **Variables** | **Univariate logistic regression analysis** | | **Multivariate logistic regression analysis** | |
| --- | --- | --- | --- | --- |
|  | ***P*** | ***OR(95%CI)*** | ***P*** | ***OR(95%CI)*** |
| Height of mother (cm) |  |  |  |  |
| < 165 | Ref. |  |  |  |
| ≥ 165 | 0.002 | 1.714 (1.222,2.404) | 0.009 | 2.303 (1.232, 4.305) |
| Pre-pregnancy BMI (kg/m2 ) |  |  |  |  |
| Normal  (18.5–24.9) | Ref. |  |  |  |
| Underweight  (<18.5) | 0.009 | 0.535(0.333,0.857) | 0.921 | 1.040 (0.477, 2.266) |
| Overweight (25.0–29.9) | ＜0.001 | 2.555(1.726,3.781) | 0.022 | 2.166 (1.119, 4.195) |
| Obesity  ( ≥ 30) | 0.002 | 3.167(1.554,6.454) | 0.046 | 3.189 (1.020, 9.968) |
| GWG rate during the first trimester (kg/week) |  |  |  |  |
| Normal | Ref. |  |  |  |
| Over | 0.025 | 1.811(1.076,3.047) | 0.267 | 1.454 (0.751, 2.814) |
| Under | 0.160 | 0.698(0.422,1.153) | 0.082 | 0.571 (0.303, 1.074) |
| GWG rate during the second trimester(kg/week) |  |  |  |  |
| Normal | Ref. |  |  |  |
| Over | 0.002 | 2.094(1.307,3.355) | 0.005 | 2.083 (1.250, 3.470) |
| Under | 0.471 | 0.662(0.216,2.033) | 0.378 | 0.566 (0.160, 2.004) |
| Abnormal blood glucose value of OGTT |  |  |  |  |
| ≤ 1 | Ref. |  |  |  |
| ≥ 2 | 0.002 | 2.348(1.358,4.062) | 0.002 | 5.267 (1.814, 15.29) |

Abbreviations: BMI: body mass index, GWG: gestational weight gain, OGTT: oral glucose tolerance test.

**Table S2.** Logistic regression analysis of risk factors for macrosomia in late pregnancy before delivery

| **Variables** | **Univariate logistic regression analysis** | | **Multivariate logistic regression analysis** | |
| --- | --- | --- | --- | --- |
|  | ***P*** | ***OR*(95%*CI*)** | ***P*** | ***OR*(95%*CI*)** |
| Maternal height (cm) |  |  |  |  |
| < 165 | Ref. |  |  |  |
| ≥ 165 | 0.002 | 1.714(1.222,2.404) | 0.034 | 1.729 (1.042, 2.869) |
| Gestational age at delivery (weeks) |  |  |  |  |
| < 40 | Ref. |  |  |  |
| ≥ 40 | ＜0.001 | 2.929(2.184,3.929) | 0.002 | 1.996 (1.284, 3.104) |
| Abnormal blood glucose value of OGTT |  |  |  |  |
| ≤ 1 | Ref. |  |  |  |
| ≥ 2 | 0.002 | 2.348(1.358,4.062) | 0.002 | 5.267 (1.814, 15.29) |
| Maternal abdominal circumference plus fundal length at pre-delivery  (cm) |  |  |  |  |
| < 139 | Ref. |  |  |  |
| ≥ 140 | ＜0.001 | 8.311(5.886,11.736) | ＜0.001 | 6.283 (3.976, 9.927) |
| Fetal BPD (cm) |  |  |  |  |
| < 10 | Ref. |  |  |  |
| ≥ 10 | ＜0.001 | 10.249(4.355,24.115) | 0.033 | 3.373 (1.103, 10.31) |
| Fetal HC(cm) |  |  |  |  |
| < 35 | Ref. |  |  |  |
| ≥ 35 | ＜0.001 | 7.567(3.828,14.96) | 0.011 | 3.473 (1.334, 9.041) |
| Fetal AC (cm) |  |  |  |  |
| < 36 | Ref. |  |  |  |
| ≥ 36 | ＜0.001 | 36.553(23.968,55.746) | ＜0.001 | 23.46 (14.81, 37.16) |

Abbreviations: OGTT: oral glucose tolerance test, BPD: biparietal diameter, HC: head circumference, AC: abdominal circumference.
